# Characterisation of *Candida parapsilosis* CYP51 as a Drug Target Using *Saccharomyces cerevisiae* as Host

**DOI:** 10.3390/jof8010069

**Published:** 2022-01-10

**Authors:** Yasmeen N. Ruma, Mikhail V. Keniya, Joel D. A. Tyndall, Brian C. Monk

**Affiliations:** 1 Sir John Walsh Research Institute, Faculty of Dentistry, University of Otago, Dunedin 9016, New Zealand; yasmeen.ruma@otago.ac.nz (Y.N.R.); mikhail.keniya@otago.ac.nz (M.V.K.); 2 School of Pharmacy, University of Otago, Dunedin 9016, New Zealand; joel.tyndall@otago.ac.nz

**Keywords:** *Candida parapsilosis*, cytochrome P450, CYP51, lanosterol 14α-demethylase, fungal pathogen, antifungal resistance, azoles, *Saccharomyces cerevisiae* expression, X-ray crystal structure, VT-1129

## Abstract

The fungal cytochrome P450 lanosterol 14α-demethylase (CYP51) is required for the biosynthesis of fungal-specific ergosterol and is the target of azole antifungal drugs. Despite proven success as a clinical target for azole antifungals, there is an urgent need to develop next-generation antifungals that target CYP51 to overcome the resistance of pathogenic fungi to existing azole drugs, toxic adverse reactions and drug interactions due to human drug-metabolizing CYPs. *Candida parapsilosis* is a readily transmitted opportunistic fungal pathogen that causes candidiasis in health care environments. In this study, we have characterised wild type *C. parapsilosis* CYP51 and its clinically significant, resistance-causing point mutation Y132F by expressing these enzymes in a *Saccharomyces cerevisiae* host system. In some cases, the enzymes were co-expressed with their cognate NADPH-cytochrome P450 reductase (CPR). Constitutive expression of CpCYP51 Y132F conferred a 10- to 12-fold resistance to fluconazole and voriconazole, reduced to ~6-fold resistance for the tetrazoles VT-1161 and VT-1129, but did not confer resistance to the long-tailed triazoles. Susceptibilities were unchanged in the case of CpCPR co-expression. Type II binding spectra showed tight triazole and tetrazole binding by affinity-purified recombinant CpCYP51. We report the X-ray crystal structure of ScCYP51 in complex with VT-1129 obtained at a resolution of 2.1 Å. Structural analysis of azole—enzyme interactions and functional studies of recombinant CYP51 from *C. parapsilosis* have improved understanding of their susceptibility to azole drugs and will help advance structure-directed antifungal discovery.

## 1. Introduction

Candidiasis is the predominant fungal infection of immunocompromised people. Although *Candida albicans* is the main causative agent of such infections, since the early 2000s there has been a marked increase in the prevalence of non-albicans-related invasive candidiasis worldwide [1]. Among the non-albicans *Candida* species, *Candida parapsilosis* has emerged as a pathogen of significant concern. It ranks in the top three among the *Candida* species most frequently isolated from immunosuppressed patients, with patient age and geographic location significantly impacting incidence [2]. 

*C. parapsilosis* is a commensal of the human skin. It is found mainly on the skin and in nails, mucosal surfaces, and the gastrointestinal tract [3,4]. Its adherence to human epithelial tissue enables ready person—person transmission of the pathogen within ICUs and elderly care facilities. A tendency to bind to artificial surfaces makes *C. parapsilosis* infection a risk for patients with central venous catheters or other implanted devices [5]. Its rapid growth in high glucose parenteral solutions [6] makes it problematic for premature neonates and extremely low birth weight infants reliant on parenteral nutrition [7]. With increasing numbers of hospitalised patients in addition to more frequent use of life support and associated interventions, contact-mediated nosocomial transmission of *C. parapsilosis* can result in major disease outbreaks in elderly care facilities [8,9,10,11] that can be lethal for vulnerable individuals [1,3,4,12]. Superficial infections caused by *C. parapsilosis* include onychomycosis [13], vaginitis in healthy women [14] and urinary tract infections associated with the use of urinary catheters [15]. Invasive infections reported include endocarditis, fungal peritonitis in peritoneal dialysis patients [16], endophthalmitis, pancreatitis, arthritis [17,18] and CNS infections [18].

Echinocandins and azole drugs are the main antifungals used for the treatment of candidiasis. According to the Clinical Practice Guideline for the Management of Candidiasis, echinocandins are the recommended first choice drugs for treating *Candida* infections in clinical settings [19]. Based on Clinical and Laboratory Standards Institute (CLSI) data, higher MIC_50_ values for micafungin, caspofungin and anidulafungin indicate that *C. parapsilosis* is the least susceptible among *Candida* species to echinocandins. This reduced susceptibility is thought to be due to natural polymorphism in the *FKS1* gene of *C. parapsilosis* [1,11,20]. In addition, prolonged in vitro exposure to echinocandins results in resistance to these drugs [21]. Due to their efficacy, azoles remain the primary alternate choice of antifungal for treating *Candida* infections, including against *C. parapsilosis* [10]. The azoles target the cytochrome P450 enzyme lanosterol 14α-demethylase (CYP51), a key enzyme in the ergosterol biosynthetic pathway. Inhibition of CYP51 not only leads to depletion of ergosterol, thus modifying the fluidity, asymmetry and integrity of the fungal cell membrane [22], but also produces toxic fecosterols [23]. 

Several recent reports have highlighted a concerning incidence of azole-resistant *C. parapsilosis* clinical isolates [11]. For example, laboratory-based surveillance in South African hospitals found that only 37% of 531 *C. parapsilosis* clinical isolates were susceptible to fluconazole (FLC) and voriconazole (VCZ) [24]. A survey of South African neonatal ICUs showed that 54% of 143 clinical isolates were FLC resistant and 14% VCZ resistant [25]. A cross-sectional study of a tertiary care hospital in Brazil reported an outbreak of candidemia caused by FLC-resistant *C. parapsilosis* strains [8], while Thomaz et al. suggested that an azole non-susceptible clone of *C. parapsilosis* was prevalent in Brazilian hospitals [26]. FLC resistant *C. parapsilosis* isolates were reported recently in Italian hospitals [11], and strains showing cross-resistance to azoles were reported in the global surveillance study performed by Pfaller et al. [27]. There is a clear need for further research on the azole susceptibility of *C. parapsilosis* and the molecular mechanisms responsible for azole resistance. 

**Figure 1:**
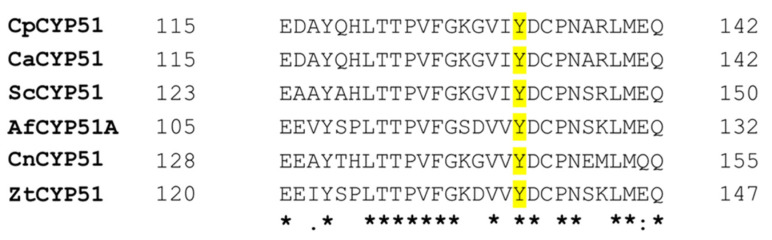
Primary sequence alignment of several fungal pathogens in the region of Y132 CpCYP51 using ClustalW. *C. albicans* CYP51 (CaCYP51), *S. cerevisiae* CYP51 (ScCYP51), *A. fumigatus* CYP51A (AfCYP51A), *C. neoformans* CYP51 (CnCYP51) and *Z. tritici* CYP51 (ZtCYP51). The tyrosine residue (Y), equivalent to Y132 in CpCYP51, is highlighted in yellow. An asterisk denotes conserved residues, a colon indicates conservative substitutions and a full stop marks semi-conservative substitutions.

Like *C. albicans*, the known mechanisms of azole resistance in *C. parapsilosis* have been related to either CYP51 or drug efflux pumps. Overexpression of *C. parapsilosis* CYP51 (CpCYP51) in the presence of azoles [28] and the point mutation Y132F in CYP51 are commonly detected in azole-resistant *C. parapsilosis* clinical isolates [11,29]. The residue homologous to CpCYP51 Y132 is conserved among most fungal pathogens (Figure 1). The development of resistance to short-tailed but not long-tailed triazoles due to replacement of the tyrosine (Y) with a phenylalanine (F) has been identified frequently in *Candida* species, notably in the CYP51s of *C. albicans* [30,31], *C. auris* [32] and *C. tropicalis* [33,34]. Other examples of this mutation include *Cryptococcus neoformans* CYP51 Y145F [35], *Aspergillus fumigatus* CYP51A Y121F [36] and *Zymoseptoria tritici* CYP51 Y137F [37]. In addition, intrinsic resistance to FLC and VCZ is partially conferred by the *Rhizopus arrhizus* CYP51 F5 Y129F [38]. 

The design of effective broad-spectrum antifungals that specifically target fungal CYP51s and are not subject to known resistance mechanisms requires understanding of the structural and biochemical features of the CYP51s of several fungal pathogens. Here we report the first detailed functional and biochemical characterisation of *C*. *parapsilosis* CYP51 and its Y132F mutant by expressing recombinant full-length hexahistidine-tagged versions of these enzymes in *Saccharomyces cerevisiae*. Such constructs will enable phenotypic screens that identify antifungals targeting these CYP51s and ultimately allow structural analysis of drug target interactions. This will assist the discovery of novel antifungals in compound libraries and help further development of drug scaffolds used to design broad-spectrum antifungals.

The tetrazole antifungals are a new generation of azoles designed to overcome the toxicity and drug interactions present in imidazole and triazoles. Improved selectivity for fungal CYP51s is obtained by reducing affinity for human CYP51 and liver drug metabolizing cytochrome P450s [39]. VT-1161, VT-1129 and VT-1598 are examples of tetrazole antifungals currently in clinical trials. The crystal structures of CYP51s in complex with VT-1161 [40,41] and VT-1598 [42] have been previously determined. In this study, we report the X-ray crystal structure of VT-1129 (quilseconazole) in complex with *S. cerevisiae* CYP51 (ScCYP51) as a surrogate to understand the molecular basis of CpCYP51 susceptibility to tetrazoles. The recombinant strains expressing CpCYP51 give comparable patterns of susceptibility to both inhibitors, and only minor differences in the interaction of VT-1161 and VT-1129 with ScCYP51 were detected. 

## 2. Materials and Methods

### 2.1. Yeast Strains and Culture Media

*S. cerevisiae* strains used in this study are listed in Supplementary Materials (Table S1). Media used for the growth and maintenance of the yeast strains were YPD/YPG comprised of 1% (*w*/*v*) Bacto yeast extract (BD Difco Laboratories, Inc., Franklin Lakes, NJ), 2% (*w*/*v*) Bacto Peptone (BD Difco), and 2% (*w*/*v*) glucose/galactose. Synthetic defined (SD) dropout medium solidified with agar was used as selective media for transformants. It consisted of 2% (*w*/*v*) glucose or galactose, 0.67% (*w*/*v*) yeast nitrogen base without amino acids (BD Difco), 1.8% (*w*/*v*) agar (Oxoid Ltd., Hampshire, UK), and 0.77 g/L uracil drop-out (QBioGene, Irvine, CA, USA) or 0.77 g/L histidine drop-out (Formedium, Norfolk, UK) or complete supplement mixture (CSM). For the MIC_80_ determinations, liquid SD medium with CSM containing 10 mM MES and 20 mM HEPES, buffered with TRIS to pH 6.8, was used. For agarose diffusion assays, this medium was solidified with 0.6% agarose [40,43].

### 2.2. Materials

Fluconazole (FLC), voriconazole (VCZ), itraconazole (ITC), posaconazole (PCZ) and amphotericin B (AmpB) were purchased from Sigma-Aldrich, Ltd. (St. Louis, MO, USA). VT-1161 and VT-1129 were prepared by MicroCombiChem (Wiesbaden, Germany) using the methods described by Hoekstra et al. [39]. Micafungin (MCF) was supplied by Astellas Pharma Inc. (Osaka, Japan). Desalted oligonucleotides (Table S2) were purchased from Integrated DNA Technologies (IDT). Codon optimised *C. parapsilosis CYP51 Y132F* and *CPR* gene sequences including sequences encoding a C-terminal hexa-histidine tag were purchased from ATUM (Newark, CA, USA). TaKaRa DNA polymerase (TaKaRa Bio Inc., Shiga, Japan) was used for colony PCR. Phusion Green Hot Start II High-Fidelity PCR master mix (2×) (ThermoFisher Scientific, Waltham, MA, USA) was used for all other PCR amplifications. PCR clean-up and DNA gel extraction were carried out using the NucleoSpin^®^ Gel and PCR clean-up kits (Macherey-Nagel, Düren, Germany). Transformation of DNA into the yeast cells was performed using an Alkali-Cation^TM^ Yeast Transformation kit (MP Biomedicals, LLC, Solon, OH, USA). Genomic DNA was extracted using a Yeast DNA Extraction Kit (Thermo Scientific, Waltham, MA, USA). DNA transformation cassettes and genes inserted at the *S. cerevisiae PDR5*, *PDR15* or *ERG11* locus were confirmed by DNA sequence analysis performed at the Genetic Analysis Services facility (University of Otago, Dunedin, New Zealand). The identity of the recombinant proteins was confirmed by mass spectrometry using an LTQ-Orbitrap hybrid mass spectrometer at the Centre for Protein Research (University of Otago, New Zealand). 

### 2.3. Construction of Recombinant Strains

The recombinant strains were constructed to overexpress *C. parapsilosis CYP51* and *CPR* genes from the *S. cerevisiae PDR5* and *PDR15* loci, respectively. The *S. cerevisiae* strain Y2494 was engineered from strain AD2Δ [43,44,45] with a *GAL1* promoter replacing the promoter of the endogenous *ERG11*. The host strain is deleted of 7 pleiotropic drug resistance (PDR) ABC transporters and the *PDR3* transcriptional regulator gene but includes the mutant gain of function *pdr1-3* transcriptional regulator that drives constitutive expression from the *PDR5* locus or from the *PDR15* locus with the *PDR5* promoter introduced [46]. The strains generated and used in this study are shown in Table 1 and Table S1 (Supplementary Materials), respectively. The primers used for the strain construction are described in Table S2. DNA transformation cassettes were introduced into the respective loci of the host strain by homologous recombination. *C. parapsilosis* wild type CYP51-6×His and CYP51-6×His Y132F were heterologously overexpressed from the *PDR5* locus. The CYP51 transformation cassettes bordered with the *PDR5*-specific arms consisted of the open reading frame (ORF) with a C-terminal hexa-histidine tag after a GGR linker, the *PGK* transcription terminator and the *HIS1* selection marker flanked with two *LoxP* sites. C-terminal hexa-histidine tagged *C. parapsilosis CPR* ORF together with a PGK transcription terminator and a selection marker (*URA3*), bordered upstream by a *PDR5* promoter sequence, was similarly overexpressed from the *PDR15* locus of the CpCYP51 and CpCYP51 Y132F expressing *S. cerevisiae* strains. The *PDR5* and the *PDR15* loci were used to overexpress both genes at comparable levels due to the pdr1-3 transcriptional regulator in the host background acting constitutively on PDRE elements in *PDR5* promoters at both loci. Recombinant yeast strains were also created by deleting the endogenous ScCYP51 from the constructed strains. To achieve this, the *HIS1* marker was first removed from the *PDR5* locus of the constructed strains using a Cre expressing plasmid-pSH69 (Euroscarf, SRD, Germany), which consists of the galactose inducible Cre recombinase, to mediate recombination between the two *LoxP* sites, thus allowing the removal of the *HIS1* [47,48]. ScCYP51 was then replaced with a Sc*His1* disruption cassette. The genes expressed from the *PDR5*, *PDR15* or the *ScERG11* loci were confirmed by DNA sequence analysis. All the recombinant strains were grown in glucose-containing media to confirm that CpCYP51-6×His and CpCYP51-6×His Y132F are functional.

### 2.4. Agarose Diffusion Drug Susceptibility and MIC_80_ Determination Assays

Azole susceptibility of recombinant strains was assessed qualitatively using agarose diffusion assays as described previously by Keniya et al. [49]. Zones of inhibition produced against the azoles were compared in test strains with MCF as an independent control. As azole drugs are fungistatic rather than fungicidal and can display a trailing effect, liquid microdilution assays were used to determine MIC_80_ values from the growth of individual recombinant yeast strains in response to each azole drug compared to the no drug control [43]. SD medium buffered to pH 6.8 was used instead of RPMI 1640 to enable efficient and reproducible growth of the *S. cerevisiae* strains [40,43,50]. The assay was carried out in 96-well microtiter plates in a total volume of 200 μL. Each well with the azole drugs serially diluted 1.4-fold was seeded with yeast cells to an initial OD_600 nm_ of 0.01 (3 × 10^4^ CFU/well). Drug-free controls and wells containing only SD media were also included. The plates were incubated at 30 °C for 48 h with shaking at 200 rpm in humidified containers to prevent evaporation. The cell density measured at 600 nm in a BioTek Synergy 2 multimode plate reader (BioTek Instruments, Winooski, VT, USA) was used to estimate the inhibitory effect of the azole drugs on the yeast strains. The experiments were carried out in triplicates for three different clones of each strain, giving a total of 9 readings [43].

### 2.5. Preparation of Crude Membranes and Western Blot Analysis of His-Tagged Recombinant Proteins

Strains deleted of the native CYP51 were used for the isolation of crude membranes and further purification. Yeast cells grown in YPD at 30 °C with shaking at 200 rpm were harvested at an OD_600 nm_ = 6–8. Harvested yeast cells were broken by bead beating, followed by isolation of crude membranes by differential centrifugation [51]. The amount of protein in the crude membranes was estimated using the Lowry method [52], with bovine serum albumin (Thermo Fisher, Waltham, MA, USA) as the standard. Samples containing 30 μg of crude membrane protein were separated by SDS-PAGE in 8% acrylamide gels at pH 8.5 using the method of Laemmli [53] and stained with Coomassie blue R250. Western blot analysis was performed by electrotransfer of the protein from the gel onto a nitrocellulose membrane (Bio-Rad, Hercules, CA, USA) and subsequent detection of immunocomplexes by chemiluminescence as described previously by Keniya et al. [43]. 

### 2.6. Protein Purification

The enzymes CpCYP51-6×His and ScCYP51-6×His were purified by Ni-NTA affinity and size exclusion chromatography (SEC) using the method described by Monk et al. [51]. Crude membranes were solubilised with 16 mM [10× critical micelle concentration (CMC)] of the detergent n-decyl-β-D-maltoside (DM) (Anatrace, Maumee, OH, USA) medium containing 10% (*w*/*v*) glycerol, 250 mM NaCl, 20 mM Tris pH 7.5, 0.5 mM PMSF and 1 EDTA-free protease inhibitor pill (Roche, Basel, Switzerland) per 200 mL of buffer. The enzymes were affinity-purified from the solubilised fraction in a HisTrap^TM^ HP 1 mL column (GE Healthcare Bio-Sciences AB, Uppsala, Sweden) using 200 mM imidazole as an eluent and further purified by SEC using a Superdex 200 10/300 GL column (GE Healthcare Life Sciences, Chalfont St Giles, UK). Buffer containing 6.4 mM DM (4 × CMC) was used for the separations as previously described [45,54]. The eluted fractions with red colour characteristic for CYP51 proteins were pooled and concentrated to 1 mL for SEC and drug binding studies using Amicon^®^ Ultracel^®^-50K Centrifugal Filters (Merck Millipore Ltd., Cork, Ireland). 

### 2.7. Spectral Characterisation and Azole Binding Assay 

The UV-visible absolute absorbance spectrum of the purified protein was obtained using an Ultrospec^TM^ 6300 pro UV-visible spectrophotometer between 250 and 600 nm. The P450 concentration of the protein was determined from the Soret band (at 417 nm) using a molar extinction coefficient value of ε_417_ = 117 mM^−1^cm^−1^ for the low spin ferric form of the protein [55]. The spectrophotometric index was determined from the ratio of absorbance at 417 nm to that at 280 nm to estimate the relative amount of heme present in the purified enzyme fraction [55]. The spin state of the purified enzyme was calculated from the absolute absorbance spectra using the equation Δ*A*_393–470/_Δ*A*_416.5–470_. A value of 0.4 indicates a 100% low spin state and value of 2.0 corresponds to 100% high spin state [56]. Type II absorbance difference spectra were recorded as a measure of azole binding [43,44] using a Cary 1 Bio UV-visible spectrophotometer. Equal volumes of 1 μM CYP51 were split into two 10 mm path UV transparent plastic cuvettes. The azoles VCZ and PCZ, dissolved in DMSO, were added in increments to the protein in the sample cuvette, and a corresponding amount of DMSO was added to the protein in the reference cuvette. Spectra between 350 and 500 nm were recorded after each addition of azole. A drug saturation curve was obtained by plotting the trough—peak absorbance changes against the drug concentration. The dissociation constant (*K_d_*) was determined by nonlinear regression (Levenberg—Marquardt algorithm) [57] by fitting the obtained data to the Hill equation Δ*A* = Δ*A*_max_ [azole]*^n^*/([azole]*^n^* + *K_d_^n^*), using GraphPad Prism 9 (GraphPad, San Diego, CA, USA), where Δ*A*_max_ is the maximum change in the absorbance, [azole] is the azole concentration and *n* is the Hill coefficient. 

### 2.8. X-ray Crystallographic Analysis

Ni-NTA affinity and SEC-purified ScCYP51-6×His was co-crystallised with VT-1129 (40 µM) using a hanging-drop vapor diffusion method at 18 °C [51]. The reservoir solution contained PEG 400 (Sigma-Aldrich) at concentrations of 44% and 45% *v*/*v* in 0.1 M glycine-NaOH buffer in the pH range of 9.3 to 9.5. The drop volume was 1 μL or 2 μL in a 1:1 ratio of reservoir solution and 20 to 30 mg/mL of protein in SEC buffer. Crystals were picked using an appropriately sized nylon loop (MiTeGen, Ithaca, NY, USA) and flash-cooled in liquid nitrogen. Data sets were collected on the MX2 beamline at the Australian Synchrotron using a Dectris EIGER 16M detector. The data were indexed and integrated using XDS [58] and scaled using AIMLESS in the CCP4 program suite [59]. Molecular replacement was carried out using Phaser-MR [60] from Phenix [61] using the structure of ScCYP51 in complex with lanosterol (PDB ID 4LXJ) as the template. Structure refinement and modelling were carried out in phenix.refine [61] and Coot [62], respectively. The ligand VT-1129 was generated from the Grade Web Server [63] and was modelled into the appropriate density in the active site. Water molecules were added into densities if at least one hydrogen bond was detected (2.5 to 3.3 Å). Figures were generated using PyMOL (Schrodinger).

### 2.9. Homology Modelling

A homology model of CpCYP51 was generated using Modeller 9.22 [64] using the structure of ScCYP51 (PDB ID: 4K0F) as a template based on a sequence alignment with multiple fungal CYP51 enzymes using the T-Coffee server [65]. The model with the lowest molpdf score was used for analysis.

## 3. Results

### 3.1. Biochemical Characterisation of Recombinant CpCYP51-6×His, CpCYP51-6×His Y132F and CpCPR-6×His 

*S. cerevisiae* strains generated in the present study are described in Table 1. *S. cerevisiae* strains were constructed expressing recombinant CpCYP51-6×His and CpCYP51-6×His Y132F, with or without a recombinant version of their cognate NADPH cytochrome P450 reductase (CpCPR-6×His) co-expressed from the *PDR15* locus and deleted of endogenous CYP51. In some strains, expression of the native CYP51 was regulated using the *GAL1* promoter, and in other strains, the native CYP51 was deleted. The viability of the resultant strains when grown in medium containing glucose as an energy source demonstrated that CpCYP51-6×His was functional. 

**Figure 2:**
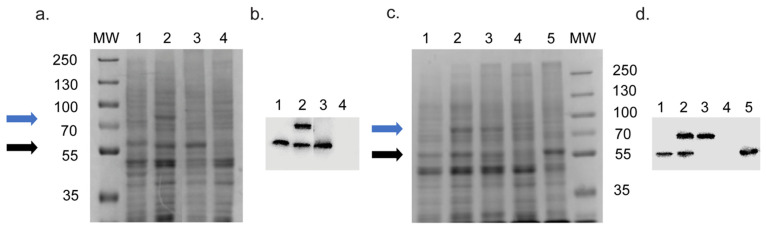
SDS-PAGE and Western blot analysis of CpCYP51-6×His, CpCYP51-6×His Y132F and CpCPR-6×His expression. (**a**,**c**) Coomassie-stained SDS-PAGE gels of crude membranes (30 μg) expressing recombinant *C. parapsilosis* proteins. (**b**,**d**) Expression of the autologous and recombinant His-tagged proteins detected in Western blots of crude membranes (30 μg/lane) decorated with mouse anti-6×His antibody visualised using ECL. (**a**,**b**) Lanes: 1. CpCYP51-6×His (Y2719), 2. CpCYP51-6×His co-expressed with CpCPR-6×His (Y2721), 3. ScCYP51-6×His (Y941), 4. Y1857 (AD2Δ). (**c**,**d**) Lanes: 1. CpCYP51-6×His Y132F (Y2714), 2. CpCYP51-6×His Y132F co-expressed with CpCPR-6×His (Y2716), 3. CpCPR-6×His (Y2717), 4. Y1857, 5. ScCYP51-6×His (Y941). Strain genotypes are given in Table 1 and Table S1 (Supplementary Material). MW: 5 µL protein molecular weight markers (PageRuler Plus Prestained Protein Ladder, Thermo Scientific), molecular weights in kDa.

The SDS-PAGE profiles of crude membrane preparations obtained from *S. cerevisiae* strains expressing recombinant CYP51s and CPR are presented in Figure 2a,c and their corresponding Western blots in Figure 2b,d, respectively. Overexpression of recombinant CYP51s from the *PDR5* locus by strains Y2719, Y2721, Y2711 and Y2712 (Figure 2a,b lanes 1 and 2) produced significantly stronger Coomassie R250-stained protein bands than the native ScCYP51 (Figure 2a,c lane 4) controlled by its endogenous promoter in the host strain (Y1857). Compared to the 62 kDa band of the ScCYP51-6×His protein constitutively overexpressed from the *PDR5* locus in strain Y941 (black arrow, MW 62 kDa, Figure 2a lane 3 and Figure 2c lane 5), wild type and mutant CpCYP51-6×His were detected at the expected size (~59.5 kDa) and at similar intensity (Figure 2a,c, lanes 1 and 2). Expression of the cognate reductase CpCPR-6×His from the *PDR15* locus gave a prominent band corresponding to the 76.8 kDa protein (blue arrow, Figure 2a,c, lane 2) and of intensity comparable to the ScCYP51-6×His control. Strain Y2717 grown on galactose also expressed a 76.8 kDa band at levels similar to the ScCYP51-6×His control (Figure 2c, lane 3 and lane 4).

The corresponding Western blots are displayed in Figure 2b,d, respectively. The native ScCYP51 was not detected on Western blot (Figure 2b,d, lane 4) as it was not His-tagged. Recombinant protein bands in the Western blots were compared quantitatively to the overexpressed standard ScCYP51-6×His protein using Image J software. The wild type CpCYP51-6×His protein (Figure 2b, lane 1) gave a relative signal of 0.70 and CpCPR-6×His (lane 2) gave a relative signal of 0.73 compared to ScCYP51-6×His protein (relative density of 1.0, lane 3). Furthermore, co-expression of the cognate reductase from the *PDR15* locus did not significantly alter the expression of CpCYP51-6×His from the *PDR5* locus (Figure 2b, lane 2). The Western blot of the strains expressing CpCYP51-6×His Y132F showed that the relative density of the CpCYP51-6×His Y132F was 0.58 (Figure 2d, lane 1), and CpCPR-6×His was 0.99 (lane 2), compared to the control protein ScCYP51-6×His (lane 5). The relative density of the reductase CpCPR-6×His was estimated to be 0.95 in the strain expressing only the reductase (lane 3), which suggests comparable reductase overexpression in this control strain and the co-expressing strain. Co-expression of the cognate reductase from the *PDR15* locus did not cause any significant change in the relative expression of CpCYP51-6×His Y132F from the *PDR5* locus (Figure 2d, lanes 1 and 2). Collectively, the Western blot data indicate that constitutive pdr1-3-dependent expression mediated by the *PDR5* promoter at the *PDR5* or *PDR15* loci is comparable and only modestly reduced by the CpCYP51-6×His Y132F mutation. 

All the recombinant enzymes, including the Y132F mutation, were confirmed using mass spectrometry of the SDS-PAGE gel bands digested with trypsin or chymotrypsin. High-percent coverage of all the three recombinant enzymes (CpCYP51-6×His, CpCYP51-6×His Y132F and CpCPR-6×His) was attained (Figure S1). The sequence coverage for the putative CpCYP51-6×His band was 67.8% with tryptic digestion, including confirmation of the C-terminal hexa-histidine tag. Chymotryptic digestion gave higher coverage of 88.3%, but this did not include the C-terminal hexa-histidine tag. Tryptic digestion of CpCYP51-6×His Y132F gave a sequence coverage of 63.09% that included the C-terminal hexa-histidine tag and confirmed the presence of F132. Chymotryptic digestion gave 93.97% sequence coverage for CpCYP51-6×His Y132F, which extended to the linker GGR only but again confirmed the presence of F132. Sequence coverage for CpCPR-6×His was 90.14% by tryptic fragmentation, including the hexa-histidine tag, and 94.49% for chymotrypsin digestion, including the linker GGR but not the hexa-histidine tag.

### 3.2. Susceptibilities of Strains Overexpressing Recombinant CYP51s to Antifungal Drugs

The susceptibilities of the recombinant strains towards azole drugs were initially screened using agarose diffusion assays. Susceptibility to the short-tailed triazoles FLC and VCZ, the long-tailed triazoles itraconazole (ITC) and posaconazole (PCZ), medium-tailed tetrazoles VT-1161 and VT-1129 and the control drugs micafungin (MCF) and amphotericin B (AmpB) was assessed. The antifungal susceptibilities of the strains expressing wild type CpCYP51-6×His or CpCYP51-6×His Y132F, with or without co-expression of the cognate NADPH-cytochrome P450 reductase (CpCPR) and with native CYP51 either expressed or deleted/repressed by the *GAL1* promoter, were examined. The strains tested are described in Table 1.

**Figure 3:**
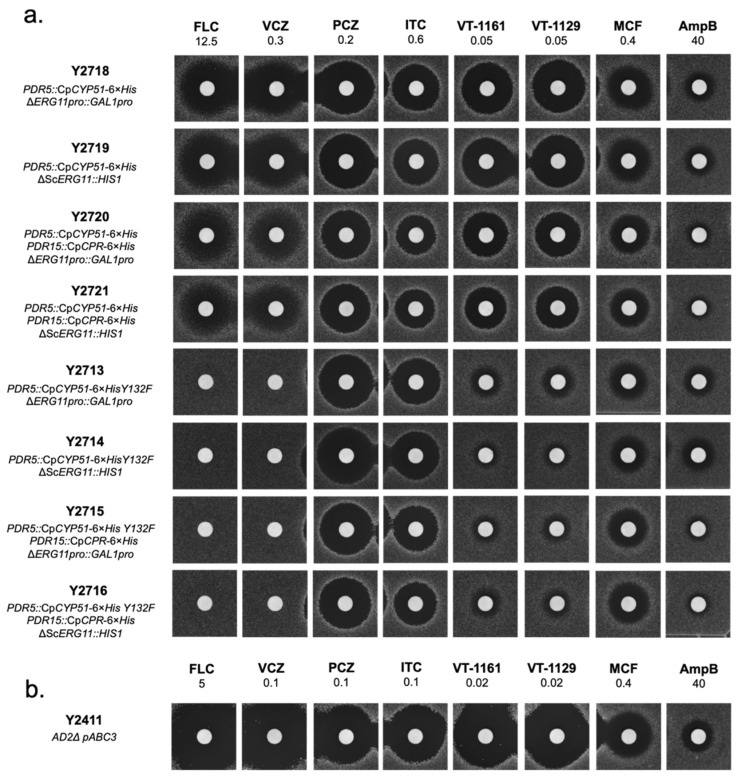
Agarose diffusion drug susceptibility analysis of host and recombinant strains to azole and control drugs. (**a**) Susceptibilities to antifungals of strains expressing recombinant CpCYP51 and CpCYP51 Y132F from the *PDR5* loci co-expressed with CpCPR from the *PDR15* loci. (**b**) Susceptibilities to the antifungals of the reference strain Y2411. The indicated amount of antifungal loaded is in nanomole per disk.

The zones of inhibition produced by the recombinant strains and the reference strain Y2411 expressing the native ScCYP51 are shown in Figure 3. The recombinant strains overexpressing either the wild type or the mutant CpCYP51 Y132F were significantly more resistant to the azoles tested than strain Y2411 (Figure 3a vs. 3b). As expected, deletion of endogenous CYP51 did not cause a visible change in susceptibility of these strains to any of the inhibitors compared to those with the native CYP51 repressed by *GAL1* promoter, i.e., similar sized zones of inhibition were obtained for Y2718 and Y2719, Y2720 and Y2721, Y2713 and Y2714, and for Y2715 and Y2716. This indicated that expression of the native ScCYP51 was repressed effectively in glucose media and that the azole resistance phenotypes of these strains were due to the functional expression of wild type CpCYP51 and CpCYP51 Y132F. The viability and drug susceptibilities of these strains confirm that the recombinant CpCYP51 is a functional drug target. Co-expression of CpCPR did not cause any change in susceptibility towards the azoles. However, a slight reduction in susceptibility to AmpB was observed, e.g., strains Y2720 and Y2721 compared to Y2718 and Y2719, respectively. The same effect was seen among the Y132F expressing strains, i.e., between Y2713 and Y2715 or Y2714 and Y2716. 

Comparison of strains Y2718–Y2721 to Y2713–Y2716 showed that the CpCYP51 Y132F protein conferred significant resistance to FLC and VCZ. With 12.5 nmole of FLC or 0.3 nmole VCZ, no inhibition zone observed with strains Y2713–Y2716 compared to the wild type strains (Figure 3a). Y2713–Y2716 required approximately three-fold more FLC than Y2718–Y2721, while around five-fold more VCZ was needed to obtain comparable sized zones of inhibition (data not shown). With similar concentrations of PCZ and ITC, equivalent sized zones of inhibition were produced by both wild type and mutant expressing strains. With 0.05 nmole of the tetrazoles VT-1161 and VT-1129, substantially reduced zones of inhibition were detected due to Y132F mutation. The control drug MCF gave equivalent sized zones of inhibition for these strains and the reference strain Y2411 (Figure 3a vs. Figure 3b). 

**Figure 4:**
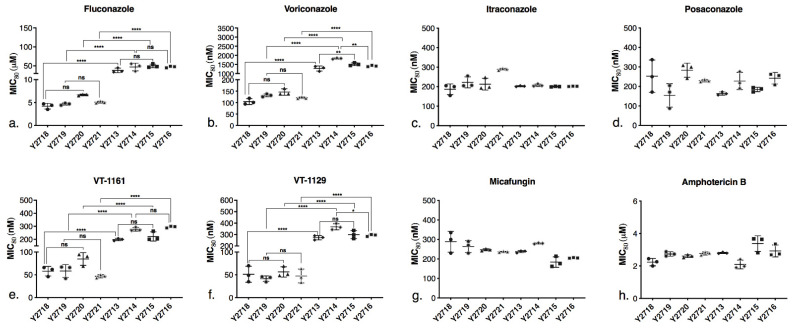
Comparison of MIC_80_ values for recombinant strains expressing CpCYP51 or CpCYP51 Y132F to antifungals. (**a**) FLC, (**b**) VCZ, (**c**) ITC, (**d**) PCZ, (**e**) VT-1161, (**f**) VT-1129, (**g**) MCF, (**h**) AmpB. Strain description is provided in Table 1. Glucose-containing SD buffered at pH 6.8 was used. The MIC_80_ values were determined in microtiter plates from the OD_600 nm_ values compared with no drug controls and were read after incubation for 48 h with shaking at 30 °C. The experiments were carried out in triplicates in three separate experiments for each strain and drug. The scatter plot is based on the average MIC_80_ values of the three separate experiments (a total of 9 measurements per strain). The horizontal line at the centre represents the mean MIC_80_, and the error bars indicate the standard error of the mean (SEM). One-way analysis of variance (ANOVA) with post hoc Tukey’s honestly significant difference (HSD) test was performed in Graph Pad Prism 9.0 to compare the difference in susceptibility between the CpCYP51 only expressing and CPR co-expressing strains, and CpCYP51 and CpCYP51 Y132F strains. The statistical significance is shown as asterisk. * *p* < 0.05, ** *p* < 0.01, **** *p* < 0.0001, ns—not significant. The *p* values for ITC, PCZ, MCF, AmpB were ns (not shown on the plot).

The MIC_80_ values of the drugs for the recombinant strains and strain Y2411 were determined (Supplementary Table S3). A scatter plot (Figure 4) compares the MIC_80_ values between the wild type and the mutant strains. All the drugs were used in nanomolar concentrations, except for FLC and AmpB which were used at micromolar concentrations. The results with MIC_80_ determinations complemented the agarose diffusion assays. All strain pairs where the native CYP51 was repressed or deleted, overexpressed from the *PDR5* locus CpCYP51 (Y2718 versus Y2719) or CpCYP51 Y132F (Y2713 versus Y2714), or those pairs which co-expressed from the *PDR15* locus the cognate reductase CpCPR (Y2720 versus Y2721 and Y2715 versus Y2716), gave comparable susceptibility to each drug tested. None of the recombinant strains expressing CpCYP51 or CpCYP51 Y132F showed increased MIC_80_ values for FLC and VCZ when the cognate reductase was co-expressed (Figure 4), consistent with the agarose diffusion assays (Figure 3a). The CpCYP51 Y132F mutation increased resistance to the triazoles FLC (Figure 4a) and VCZ (Figure 4b) by 10- and 12-fold, respectively. For example, strain Y2719, which heterologously expresses recombinant CpCYP51, gave an MIC_80_ value for VCZ = 131 nM while Y2714, which heterologously expresses CpCYP51 Y132F, gave an MIC_80_ = 1820 nM. The strains Y2721 and Y2716, which co-express CpCPR with CpCYP51 or CpCYP51 Y132F, gave comparable MIC_80_s of 121 nM and 1410 nM, respectively.

In contrast, the susceptibility patterns of the strains expressing CpCYP51 or CpCYP51 Y132F for the long-tailed triazoles ITC and PCZ were not significantly affected by the Y132F mutation or co-expression of CpCPR or (Figure 4c,d). The MIC_80_ values of the CpCYP51 and CpCYP51 Y132F overexpressing strains for ITC and PCZ were in the range of 150–300 nM and were thus comparable to Y2411 (Table S3). The susceptibilities of strains Y2713–Y2716, which overexpress CpCYP51 Y132F, to the tetrazoles VT-1161 and VT-1129 were intermediate between those of the short- and long-tailed azoles, i.e., the mutant enzyme conferred up to an eight-fold reduction in susceptibility to these azoles. The CpCYP51 Y132F strains (Y2713–Y2716) were three- to eight-fold less susceptible to VT-1161 (Figure 4e) and VT-1129 (Figure 4f) than the CpCYP51 strains (Y2713–Y2716). As expected, all recombinant strains expressing CpCYP51 or CpCYP51 Y132F and strain Y2411 showed comparable susceptibilities to the control drug MCF (Figure 4g and Table S3). Comparable susceptibility to AmpB suggests that the expression of the constructs did not alter significantly the ergosterol content in the recombinant strains compared to the control strain Y2411 (Figure 4h and Table S3). 

### 3.3. Spectral Characteristics and Azole Binding of Purified C. parapsilosis CYP51

**Figure 5:**
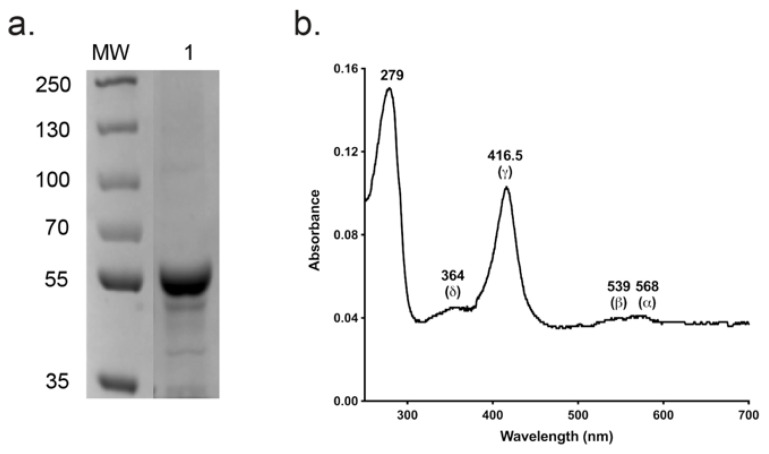
Analysis of SEC-purified CpCYP51-6×His. (**a**) Coomassie-stained SDS-PAGE profile of purified wild type CpCYP51-6×His (~59.5 kDa). MW: 5 µL protein molecular weight markers (PageRuler Plus pre-stained protein ladder, Thermo Scientific). (**b**) Absolute absorbance spectrum of the SEC-purified CpCYP51-6×His.

The absolute absorbance spectrum of Ni-NTA and SEC-purified CpCYP51-6×His (Figure 5a) exhibited α, β, Soret (γ) and δ spectral bands at 568, 539, 416.5 and 364 nm, respectively (Figure 5b). A spectrophotometric index of 0.7 (A_416.5 nm_/A_280 nm_) confirmed that the enzyme was of high purity. The ratio ΔA_393–470 nm/_ΔA_416.5–470 nm_ equal to 0.34 implied that the enzyme was completely in the low spin state [55]. The similar intensities of the α-568 nm and β-539 nm bands and absence of an absorption peak in the 650 nm region showed the CpCYP51-6×His was primarily in the oxidised enzymatically active form [56]. 

**Figure 6:**
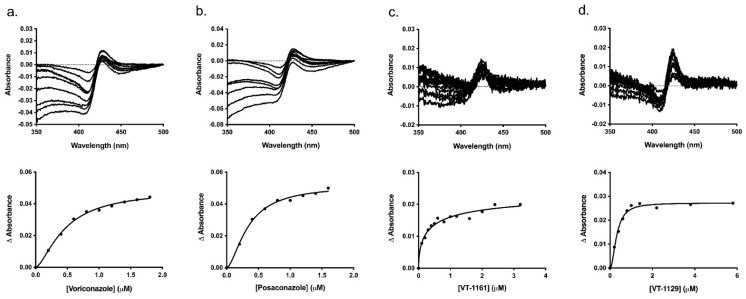
Type II binding spectra and binding curves of CpCYP51-6×His. The curves with best fit were obtained using Hill equation for the binding of the azoles to CpCYP51-6×His. (**a**) VCZ, (**b**) PCZ, (**c**) VT-1161, (**d**) VT-1129. VCZ was dissolved in Milli-Q water. PCZ, VT-1161 and VT-1129 solutions were prepared in DMSO.

Typical type II binding spectra were obtained on titration of Ni-NTA-purified CpCYP51-6×His (1 μM) with VCZ, PCZ, VT-1161 and VT-1129. The difference in absorbance (∆*A*_max_) was almost two-fold less on binding to the tetrazoles VT-1161 and VT-1129 compared to that of the triazoles VCZ and PCZ, with peaks shifted from 429–427 nm for triazole binding to 423–425 nm for tetrazole binding and the troughs shifted from 411–407 nm for triazole binding to 411–405 nm for tetrazole binding (Figure 6 and Table 2). The broad troughs show that the enzyme’s heme iron was completely in the low spin state before exposure to the inhibitors [50]. The narrower trough for the binding of VT-1129 compared with VT-1161 indicated significant differences in the electronic configurations of the heme on the binding of these closely structurally related tetrazoles. The corresponding saturation curves were plotted by fitting in to the Hill equation (Figure 6), and the binding parameters (*K_d_* and Hill coefficient) are tabulated in Table 2. Dissociation constant (*K_d_*) values significantly lower than the enzyme concentration showed that all the drugs bound tightly to the enzyme [66].

### 3.4. X-ray Crystallographic Analysis of ScCYP51-6×His in Complex with VT-1129

**Figure 7:**
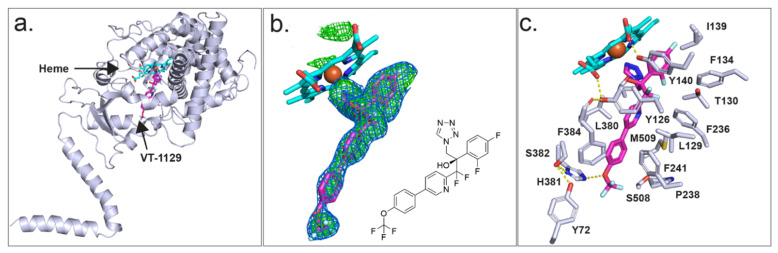
Crystal structure of ScCYP51-6×His in complex with VT-1129. (**a**) Full-length structure of ScCYP51-6×His in complex with VT-1129. (**b**) OMIT map for VT-1129 (Fo-Fc map (green mesh) contoured at 3σ; 2Fo-Fc map (blue mesh) contoured at 1σ). The Fo-Fc map was calculated using Fcalc refined from coordinates with no ligand at the active site. The 2Fo-Fc map was calculated following the final refinement. The chemical structure of VT-1129 is shown at the bottom right-hand corner. (**c**) Residues within 4 Å of VT-1129 are shown as white sticks. H-bonds are presented as yellow dashed lines. The ScCYP51 cartoon is white, VT-1129 is shown as magenta and the heme is depicted as cyan sticks with the heme iron as an orange sphere. Nitrogen atoms are blue and oxygen atoms are red.

As we are currently unable to obtain diffracting crystals of CpCYP51-6×His, we have used instead ScCYP51-6×His as a surrogate to investigate the binding of azole drugs. X-ray crystal structures were previously obtained for ScCYP51 in complex with the azole drugs FLC (PDB ID: 4WMZ), VCZ (PDB ID: 5HS1), ITC (PDB ID: 5EQB) and VT-1161 (PDB ID: 5UL0), but there was no crystal structure for a CYP51 in complex with VT-1129. The X-ray crystal structure of full-length ScCYP51-6×His in complex with VT-1129 (PDB ID: 7RYX) (Figure 7a) was obtained at a resolution of 2.1 Å for Ni-NTA affinity and SEC-purified protein. The enzyme was crystallised using 44% PEG 400, 0.1 M glycine at pH 9.5. The statistics for the X-ray data collection and structure refinement are shown in Supplementary Table S4. Molecular replacement was carried out using ScCYP51-6×His in complex with lanosterol (PDB ID: 4LXJ) as a template [51]. The structure includes the full protein sequence from isoleucine 7 (I7) to the first histidine of the C-terminal hexa-histidine tag, most of the N-terminal membrane-associated helix, the transmembrane domain and the catalytic domain, including the fungus-specific loop. 

Strong electron density within the active site allowed modelling of VT-1129 (Figure 7b). The ligand occupied the sixth axial (distal) coordination site with the heme iron via either the N-3 or N-4 atom of the tetrazole ring, at a distance of 2.1 Å. Modelling from the electron density obtained was unable to distinguish between the two possible orientations of the tetrazole ring. There are 20 residues within 4 Å of VT-1129 (Figure 7c). These are Y72 from Helix A′, Y126, L129, T130, F134 I139, and Y140 from substrate recognition site 1 (SRS1); F236, P238 and F241 in Helix FF′; G310, G314, G315, T318 from SRS4; L380, H381, S382 and F384 from SRS5; and S508 and M509 from SRS6. For VT-1129, F134 in the BC loop and F241 in the neck between the substrate entry channel and the active site in yeast were detected in proximity to this ligand rather than F506 and T507 for VT-1161.

Unexpectedly, the water mediating the hydrogen bond network previously seen between the heme ring C propionate carboxyl (RCC), the Y140 residue and the tertiary hydroxyl group in VT-1161 [40] or the triazoles FLC or VCZ [50] was not observed in the complex with VT-1129, although there is some evidence of density at the position of the water. In the absence of water, the Y140 hydroxyl forms a short hydrogen bond only with the heme RCC. The tail of VT-1129 lies in the substrate entry channel. The formation of a weak 3.2 Å hydrogen bond between the oxygen atom of the trifluoromethoxy group and the imidazole of the residue H381 was excluded due to directionality requirements (Figure 7c).

### 3.5. Analysis of CpCYP51 Model in Complex with Triazoles and Tetrazoles

**Figure 8:**
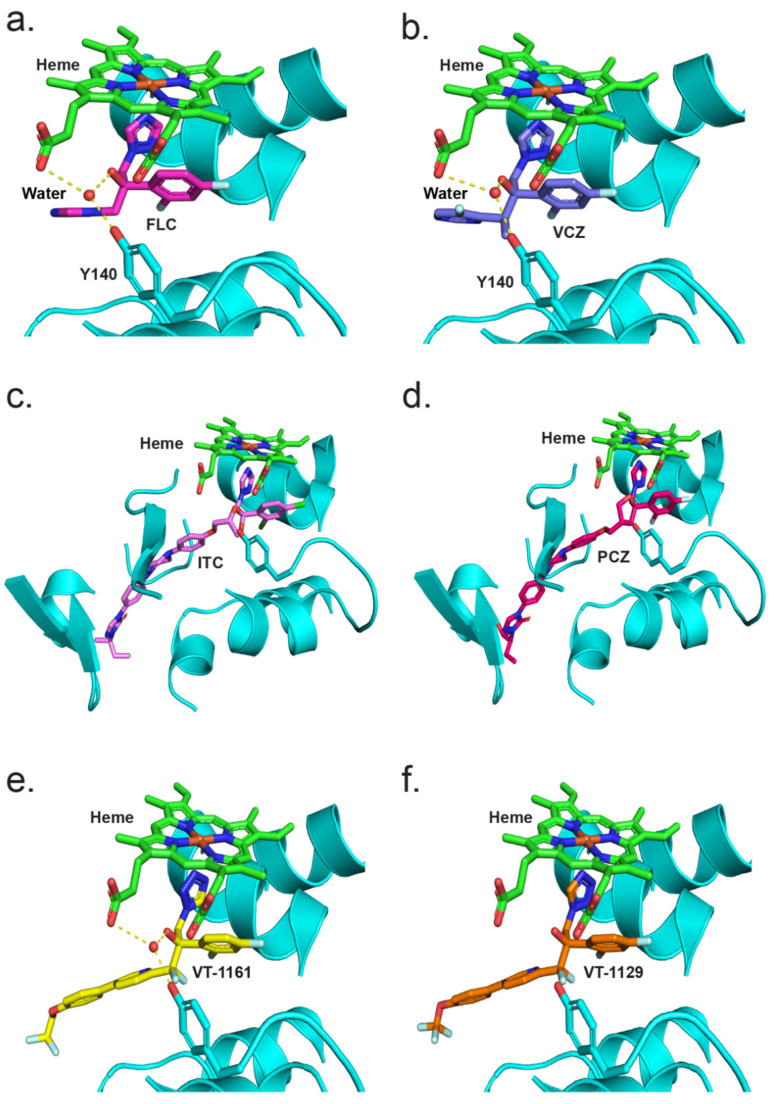
The CpCYP51 model (cyan cartoon) superimposed with ScCYP51 structures complexed with (**a**) FLC (PDB ID: 4WMZ), (**b**) VCZ (PDB ID: 5HS1), (**c**) ITC (PDB ID: 5EQB), (**d**) PCZ (PDB ID: 6E8Q), (**e**) VT-1161 (PDB ID: 5UL0), and (**f**) VT-1129 (PDB ID: 7RYX). Only ligands and selected water molecules from known ScCYP51 structures are shown.

To better understand the interaction of azoles with CpCYP51, a homology model of CpCYP51 was obtained using ScCYP51 (PDB ID: 4K0F) as a template. The interactions between CpCYP51 and ligands can be gleaned by the superimposition of the known liganded structures with the model (Figure 8). Looking at the binding of FLC (Figure 8a) [44], the water molecule (743) present in the structure of ScCYP51 in complex with FLC is able to make a similar hydrogen bond network (2.7 to 3.3 Å) between the hydroxyl group of FLC, the hydroxyl of Y132 and the heme RCC. Similar interactions can be seen for VCZ (Figure 8b) and VT-1161 (Figure 8e). ITC (Figure 8c) and PCZ (Figure 8d) are also shown in the active site of CpCYP51 but do not form a hydrogen bond network as the tertiary alcohol is replaced with a bulkier dioxolane/oxolane group. As discussed above, VT-1129 (Figure 8f) lacked sufficient density to model the water, but it can be assumed to be partially present.

## 4. Discussion

### 4.1. Recombinant Full-Length CpCYP51 Is Functionally Expressed in S. cerevisiae

Heterologous overexpression of functional, full-length hexa-histidine tagged *C. parapsilosis* CYP51s, followed by co-expression with their redox partner CPR, was successful in our *S. cerevisiae* expression system as were the previously published *C. albicans* and *C. glabrata* CYP51s [43]. The constitutive overexpression of CpCYP51-6×His and CpCYP51-6×His Y132F from the *PDR5* locus of a *S. cerevisiae* host strain deleted of seven ABC transporters has provided a stable platform to characterise these enzymes functionally and biochemically. Due to the deletion of the Pdr3 transcriptional regulator and seven ABC transporters in our host strain, which prevents drug transport from the cells by efflux pumps, the susceptibility to azole drugs of the resultant recombinant yeast strains was primarily due to the constitutive overexpression of CYP51s from the *PDR5* locus. As constitutive overexpression was controlled by the mutant gain of function pdr1-3 transcriptional regulator, azole drug concentrations had no effect on the expression of the efflux pumps or CYP51 in our host system. In addition, the recombinant wild type CpCYP51 or mutant CpCYP51 Y132F enzymes were shown to sustain growth when the native CYP51 was deleted or their *GAL* promoter-dependent expression was blocked in presence of glucose. 

Functional CpCYP51-6×His was demonstrated by showing type II binding of VCZ and PCZ. Both drugs caused a red shift in the Soret peak of CpCYP51 from 417 nm to 420–422 nm. Type II binding of these drugs gave dissociation constants (*Kd*) indicative of tight 1-1 binding and Hill coefficients in the same range as obtained previously with *S. cerevisiae*, *C. albicans* and *C. glabrata* CYP51-6×His constructs expressed in the same *S. cerevisiae* host system [43], except for VT-1161 binding where a Hill co-efficient <1 was found.

### 4.2. Differential Susceptibility of Wild Type CpCYP51 and CpCYP51 Y132F to Triazole Drugs

Drug susceptibility assays demonstrated that CpCYP51 Y132F significantly increased resistance to the short-tailed triazoles FLC and VCZ, gave a more modest increase in resistance to the medium-tailed tetrazoles VT-1161 and VT-1129, and showed no significant change in susceptibility to the long-tailed triazoles ITC and PCZ. These differences in susceptibility can be explained by considering the substantial conservation of amino acid residues within the ligand binding pocket (LBP) of fungal CYP51s.

**Figure 9:**
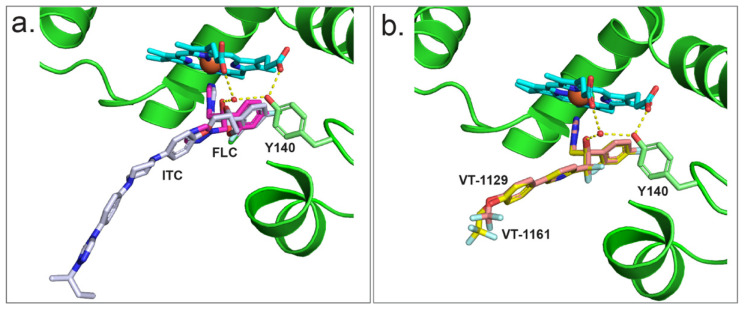
(**a**) Binding of FLC (PDB ID: 4WMZ) (magenta stick) and ITC (PDB ID: 5EQB) (grey stick) in the active site of ScCYP51. (**b**) Binding of VT-1161 (PDB ID: 5UL0) (yellow stick) and VT-1129 (PDB ID: 7RYX) (salmon stick) in the active site of ScCYP51. The ScCYP51 cartoon is green, and heme is depicted as cyan stick with the heme iron as orange sphere. Nitrogen atoms are blue and oxygen atoms are red. H-bond network involving the water molecules (small red sphere), the OH group of Y140 and the heme RCC and RCD are shown as yellow dashed lines.

Crystal structures of full-length CYP51 of *S. cerevisiae* [44,50,51,67] and two prominent fungal pathogens *C. albicans* and *C. glabrata* [54], as well as the catalytic domains of the *C. albicans* and *A. fumigatus* CYP51s [42] in complex with various azoles, are available in the PDB. From these structures, drug–protein interactions, the effects of point mutations on these interactions, key water molecules and constraining interactions between the heme and the ligand have been identified [40,44,50,51,67]. The CYP51 LBP comprises a heme at the base of the active site, a substrate entry channel and a putative product exit channel [68]. The crystal structures of full-length ScCYP51, CaCYP51 and CgCYP51 have identified up to 50 amino acid residues that contribute to the surface of the LBP [54]. Comparison of the CpCYP51 homology model with ScCYP51, CaCYP51 and CgCYP51 showed that the amino acid residues within the LBP of these enzymes are largely conserved. The residue structurally aligned with ScCYP51 Y140, equivalent to Y132 in CaCYP51 and CpCYP51, is therefore expected to be involved in a water-mediated hydrogen bond network that preferentially affects the binding in the active site of tertiary alcohol-containing, short-tailed triazoles such as FLC and VCZ, as well as the medium-tailed tetrazole VT-1161 (Figure 9). Similarly, the CpCYP51 Y132F mutation confers resistance to the short-tailed azoles FLC and VCZ and the medium-tailed tetrazole congeners VT-1161 and VT-1129 but not long-tailed azoles PCZ and ITC. Hence, the differential susceptibility to short-, medium- and long-tailed azoles due to the CpCYP51 Y132F mutation could be explained by analogy with the crystal structures of ScCYP51 Y140F in complex with such azoles. 

The resistance mechanism can be interpreted by comparing the crystal structures of ScCYP51 in complex with FLC and VCZ with the structures of the Y140F mutant with FLC (PDB ID: 4ZDZ) and VCZ (PDB ID: 4ZE0), respectively [44]. The water-mediated hydrogen bond network formed between the tertiary alcohol of FLC and VCZ with the Y140 residue was important for the binding of these triazoles, e.g., FLC binding (Figure 9a). Substitution of tyrosine by phenylalanine (Y140F) not only abolished the water-mediated hydrogen bond network but also a hydrogen bond interaction between the tyrosine hydroxyl and the heme RCC, leading to weaker binding and resistance. In addition, VCZ moves 0.5 Å closer to helix I because of the Y140F mutation [50]. In contrast, susceptibility to long-tailed triazoles ITC and PCZ was not affected by the ScCYP51 Y140F mutation. These drugs have a 1,3-dioxolane or 3-oxolane moiety, respectively, replacing the tertiary hydroxyl group in FLC and VCZ. The crystal structures of ScCYP51 Y140F bound to ITC and PCZ (PBD IDs: 4ZDY and 4ZE1, respectively) have shown that there is no water-mediated hydrogen bond network. Moreover, additional interactions of the ITC/PCZ tail within the substrate entry channel that stabilise the binding of these ligands to the enzyme help render the Y140F mutation ineffective (Figure 9a). 

### 4.3. The CpCYP51 Y132F Mutation Confers Significant Resistance to Tetrazoles

Each recombinant strain expressing CpCYP51 showed equivalent susceptibility to VT-1161 and VT-1129 but with strains expressing recombinant CpCYP51 being four to six-fold more susceptible than strains expressing recombinant CpCYP51 Y132F.

Figure 9b shows a comparison between the crystal structures of ScCYP51 in complex with VT-1161 and VT-1129. Both drugs bind to CYP51 in a similar fashion. VT-1161 interacts with the heme iron through the tetrazole N3 atom and, similar to short-tailed azoles, the tertiary hydroxyl group of VT-1161 forms a water-mediated hydrogen bond network with the residue Y140 which also forms a hydrogen bond with the heme RCC. The crystal structure of the CaCYP51 catalytic domain also forms a similar network with VT-1161 (PDB ID: 5TZ1) [41]. Disruption of this by Y140F/Y132F mutations results in reduced susceptibility to VT-1161 in ScCYP51/CaCYP51. Therefore, the resistance to VT-1161 conferred by the expression in yeast of recombinant CpCYP51 Y132F can be considered to be equivalent to that conferred by overexpression of recombinant ScCYP51 Y140F in yeast. The ScCYP51 structure in complex with VT-1129 lacked the water-mediated hydrogen bond network involving the VT-1129 tertiary alcohol and Y140, but its presence cannot be excluded. 

Hargrove et al. previously highlighted the significance of a H-Bond (2.8 Å) between the trifluoroethoxyphenyl oxygen of VT-1161 and the imidazole side chain of H377 residue in the structure of the CaCYP51 catalytic domain that would strengthen the inhibitory activity of VT-1161 [41]. Further, the homologous residue H374 in *A. fumigatus* CYP51B was suggested to contribute significantly to binding the experimental tetrazole VT-1598 [42]. However, our studies with full-length ScCYP51 show that neither VT-1161 [40,41] nor VT-1129 are likely to form hydrogen bonds with the homologous residue H381. 

## 5. Conclusions

The expression of functional, full-length recombinant *C. parapsilosis* CYP51 and CYP51 Y132F in *S. cerevisiae* is expected to enable phenotypic and biochemical screens that can identify antifungals targeting these CYP51s from an increasingly important fungal pathogen. This will help the discovery of novel antifungals in compound libraries and assist the further development of drug scaffolds used to design broad-spectrum antifungals. The yeast strains developed here have allowed for study of the differential behaviour of the CYP51s to both established and novel antifungals, indicating the potential of these strains as screening tools. Co-expression of CpCYP51 with its cognate CPR also provides opportunity for in vitro biochemical assays using fluorescent substrates such as BOMCC [69]. The present study also provides convenient and functional CYP51 templates to explore substrate binding, drug binding and inhibition, and to elucidate how mutations confer resistance. Our interpretation of the resistance mechanism detected through phenotypic and biochemical analysis of recombinant CpCYP51 and the CpCYP51 Y132F mutant expressed in *S. cerevisiae* host has broadened our understanding of structurally related mutations. The 10- to 12-fold increase in resistance to the short-tailed azoles conferred by CpCYP51 Y132F mutation was reduced to six-fold for VT-1161 and its congener VT-1129, indicating that the impact of water-mediated hydrogen bond network involving the tertiary alcohol in FLC, VCZ and VT-1161 was partially reduced by the medium-length tails in VT-1161 and VT-1129. 

## Figures and Tables

**Table 1:** List of strains prepared in the present study.

Strain	Strain Description
Wild type CpCYP51-6×His expressing strains	
Y2718	*PDR5::*Cp*CYP51*-6×*His*, ΔSc*CYP51pro::GAL1pro*
Y2719	*PDR5::*Cp*CYP51*-6×*His*, ΔSc*CYP51::HIS1*
Y2720	*PDR5::*Cp*CYP51*-6×*His*, *PDR15::*Cp*CPR*-6×*His*, ΔSc*CYP51pro::GAL1pro*
Y2721	*PDR5::*Cp*CYP51*-6×*His*, *PDR15::*Cp*CPR*-6×*His*, ΔSc*CYP51::HIS1*
Y132F mutant CpCYP51-6×His expressing strains	
Y2713	*PDR5::*Cp*CYP51*-6×*His Y132F*, ΔSc*CYP51pro::GAL1pro*
Y2714	*PDR5::*Cp*CYP51*-6×*His Y132F*, ΔSc*CYP51::HIS1*
Y2715	*PDR5::*Cp*CYP51*-6×*His Y132F*, *PDR15::*Cp*CPR*-6×*His*, ΔSc*CYP51pro::GAL1pro*
Y2716	*PDR5::*Cp*CYP51*-6×*His Y132F*, *PDR15::*Cp*CPR*-6×*His*, ΔSc*CYP51::HIS1*
CpCPR-6×His only expressing strain	
Y2717	*PDR15::*Cp*CPR*-6×*His*, ΔSc*CYP51pro::GAL1pro*

**Table 2:** Type II binding of azoles to CpCYP51-6xHis.

Triazole	∆*A*_max_	λ_peak_	λ_trough_	Hill Number	*K_d_* (μM)
Voriconazole	0.049	429 to 428	411 to 407	1.53	0.46 ± (0.03)
Posaconazole	0.052	428 to 427	411 to 407	1.63	0.34 ± (0.03)
VT-1161	0.025	424 to 423	411 to 405	0.58	0.38 ± (0.01)
VT-1129	0.027	425 to 423.5	409.5 to 406	1.96	0.32 ± (0.02)

Values in brackets indicate standard errors.

## Data Availability

Refined X-ray crystal structures with accession code 7RYX have been lodged in the Protein Data Bank.

## Supplementary Materials

The following are available online at https://www.mdpi.com/article/10.3390/jof8010069/s1, Table S1: Saccharomyces cerevisiae parental strains used in the study, Table S2: Oligonucleotides used in the study, Table S3: MIC80 values for strains overexpressing CpCYP51 or CpCYP51 Y132F with/without CpCPR, Table S4: Data collection and refinement statistics for ScCYP51-6×His in complex with VT-1129, Figure S1: Identification of the recombinant proteins by mass spectrometry.
