# Supplementary material for: Characterisation of Candida parapsilosis CYP51 as a Drug Target Using Saccharomyces cerevisiae as Host
Source: J Fungi (Basel). 2022 Jan 10;8(1):69. doi: 10.3390/jof8010069 (PMC8781857; doi:10.3390/jof8010069)
Supplement: Supplementary file 1 [file jof-08-00069-s001.zip › jof-1528068-supplementary.pdf]

Table S1. *S. cerevisiae* parental strains used in the study

Strain	Genotype	Source	Reference
**ADΔ**	MATα *PDR1-3 ΔYOR1::hisG ΔSNQ2::hisG ΔPDR3::hisG ΔPDR10::hisG ΔPDR11::hisG ΔYCF1::hisG ΔPDR55::hisG ΔPDR15::hisG Δura3 Δhis*AD124567 *ΔPDR5::hisG ΔPDR15::hisG, Δura3*	Lamping et al, 2007	[1]
**Y941**	ADΔ, Δpdr5::ScCYP51-6xHIS-URA3	Monk et al, 2014	[2]
**Y1857**	AD2Δ (ADΔ, ΔHIS1::dpl200)	Sagatova et al, 2016	[3]
**Y2411**	AD2Δ, ΔPDR5::URA3	E. Lamping	
**Y2494**	AD2Δ, ΔCYP51pro::GAL1pro	Monk et al, 2019	[4]

Table S2. Oligonucleotides used in the study

**A. Construction of CpCYP51-6×His Y132F cassette by 3-fragment fusion for expression from the *PDR5* locus**		
1.Amplification of CpCYP51-6×His Y132F ORF from plasmids received from ATUM		
Forward primer	PDR5us-CpCYP51 Y132F_f (52)	CCGCTCGTTCGAAAGACTTAATTAAAAAATGGCTTTGGTGGACTTGGCGTTG
Reverse primer	6 HisStop_r (26)	CGAATTTAATGATGATGGTGATGATG
2. Amplification of upstream fragments from gDNA of selected strain		
Forward primer	PDR5Fv3_f (23)	TCGCATTCTGCGCCTTCGAGCAC
Reverse primer	pABC3-PacI_r (31)	CATTTTTTAATTAAGTCTTTCGAACGAGCGG
3. Amplification of downstream fragment from gDNA of selected strain		
Forward primer	6HisStop-f (26)	CATCATCACCATCATCATTAAATTCG
Reverse primer	PDR5_186DS_r (25)	TTCGGACATTGAACTTTGATTTATC
**B. For wild type strain construct reversion**		
Forward primer	CpCyp51_Y132_f (31)	GTAAAGGTGTGATCTACGATTGTCCTAACGC
Reverse primer	CpCyp51_Y132_r (31)	GCGTTAGGACAATCGTAGATCACACCTTTAC
**C. Construction of CpCPR-6×His cassette by 3-fragment fusion for expression from the *PDR15* locus**		
1. Amplification of CpCPR-6×His ORF from plasmids received from ATUM		
Forward primer	PDR5us-CpCPR (54)	CCGCTCGTTCGAAAGACTTAATTAAAAAATGGCTTTAGATAGACTAGATCTTAC
Reverse primer	6 HisStop -r (26)	
2. Amplification of upstream fragments from gDNA of selected strain		
Forward primer	PDR15us_f (25)	GTCACGCCGCCGAACTGCAGCGCGC
Reverse primer	pABC3-PacI_r (31)	CATTTTTTAATTAAGTCTTTCGAACGAGCGG
3. Amplification of downstream fragment from gDNA of selected strain		
Forward primer	Not1-6xHis (34)	GGCGGCCGCCATCATCACCATCATCATTAAATTC
Reverse primer	PDR15DS_r (25)	GATGGAATAATCCAGTTCGACTCTG
**D. Amplification of ScHIS1 disruption cassette from gDNA of selected strains**		

Forward primer	ScErg11_US-773_f (24)	GCAACAATGGGCGGTTGTTTAGAG
Reverse primer	ScErg11DS346_r (25)	GACTGCTTTATTTCtGCTTGGCCTG

*f and r denotes forward and reverse primer respectively. Number of nucleotides is shown in brackets.

Table S3. MIC_{80} values for strains overexpressing CpCYP51, CpCYP51 Y132F with/without CpCPR

**Strains**	**MIC_{80}**							
	**FLC (μM)**	**VCZ (nM)**	**ITC (nM)**	**PCZ (nM)**	**VT-1161 (nM)**	**VT-1129 (nM)**	**MCF (nM)**	**AmpB (μM)**
**Y2411**	2.4±0.08	41.7±1.6	128±12	157±3.4	27.1±0.5	26.7±0.2	212±15	2.69±0.2
**Y2718**	4.2±0.4	104±7.5	187±16	253±48	58±6.1	51.3±10	288±31	2.24±0.13
**Y2719**	4.65 ±0.13	131±3.6	222±16	188±9.6	58±8	41.6±3.5	263±18	2.74±0.08
**Y2720**	6.67 ±0.09	146±8.3	213±18	283±21	84.7±8	56.5±6.5	247±3.0	2.58±0.03
**Y2721**	4.97 ±0.03	121±2.03	291±3.3	229±3.5	46.8±2	47.5±8.8	237±1.5	2.77±0.05
**Y2713**	38.2±3.4	1280±81	202±1.4	164±4.7	199±5.4	264±24	237±3.2	2.8±0.02
**Y2714**	47.2±5.8	1820±18	207±3.6	242±17	277±8.1	367±16	280±1.5	2.09±0.2
**Y2715**	50±2.8	1540±44	201±0.7	179±0.5	222±20	299±21	184±16	3.38±0.3
**Y2716**	49±0.01	1410±20	202±0.13	228±25	295±3.8	293±5.2	205±0.9	2.9±0.2

MIC_{80}s are shown as the mean values ± SEM for 3 separate clones of each strain using data obtained in triplicate measurements from at least 3 different experiments (a total of 9 measurements per strain).

Table S4. Data collection and refinement statistics for ScCYP51-6×His in complex with VT-1129

**Protein**	ScCYP51-6×His
**Ligand**	VT-1129
**PDB ID**	7RYX
**Data collection**	
Diffraction source	MX2
Wavelength (Å)	0.9537
Space group	P 1 2_1 1
Cell dimensions	
a, b, c (Å)	78.4, 67.98, 80.3
α, β, γ (°)	90, 99.38, 90
Total reflections	279113
Unique reflections	48767
Resolution (Å)	2.1 - 45.19 (2.16- 2.10)
R_{merge}	0.086 (0.720)
I / σ I	8.6 (1.7)
Completeness (%)	99.8 (99.9)
Redundancy	5.7 (6.0)
$CC_{1/2}$	0.996 (0.893)
**Refinement**	
Resolution (Å)	2.1
No. reflections	48681
Rwork / Rfree	0.2336/0.2684
No. of atoms	
Protein	4271
Ligand/ion	79
Water	41
B-factors (Å^2)	
Protein	61.69
Ligand/ion	49.68
Water	52.63
R.m.s. deviations	
Bond lengths (Å)	0.008
Bond angles (°)	0.94
Ramachandran favored (%)	95.44
Ramachandran allowed (%)	3.99
Ramachandran outliers (%)	0.57
Rotamer outliers (%)	0.65
Clashscore	5.79

A. Mass spectrometry result of CpCYP51-6×His

Digestion of CpCYP51-6×His with trypsin

MALVDLALHGYNYFMTLSTLQQFGLLVFAPFIYNIIWQLLYSLRKDRVPLVFYWIPWVGSAVSYGQDPYGFFEQCREKYGDLF
SFVMLGRVMTVYLGPKGHEFVFNAKLSDVSAEDAYQHLTTPVFGKGVIYDCPNARLMEQKKFAKTALTTDSFRRYVPLIRGEI
LDYFTKSKVFNMKKQKSGVVDVLQSQPEITIFTASRSLLGEAMRKRFDASFAQLYADLDKGFTPINFVFPHLPLPHYWKRDAA
QQKISETYMTEIARRRETGDIDENRDLIDSLLVNSTYKDGVKMTDQEIANLLIGVLMGGQHTSATTSAWFLLHLAEKPQLQDE
LYQEVLNALSGKGGNLDDLSYEDLQQMPLVNNTIKETLRLHMPLHSIFRKVVSPLVVPNTKYIVPRGHHVLVSPGYAHTNERF
YKDASDFNPHRWDESASTNDAGEVDYGFGKVSKGVSSSYLPFGGGRHRCIGEQFAYVQLGTILTTFVYNLKWKLANGKVPDVD
YTSMVTLPQHPAEIVWEKRDTCVIGGRHHHHHH

Protein sequence coverage is 67.8%

Digestion of CpCYP51-6×His with chymotrypsin

MALVDLALHGYNYFMTLSTLQQFGLLVFAPFIYNIIWQLLYSLRKDRVPLVFYWIPWVGSAVSYGQDPYGFFEQCREKYGDLF
SFVMLGRVMTVYLGPKGHEFVFNAKLSDVSAEDAYQHLTTPVFGKGVIYDCPNARLMEQKKFAKTALTTDSFRRYVPLIRGEI
LDYFTKSKVFNMKKQKSGVVDVLQSQPEITIFTASRSLLGEAMRKRFDASFAQLYADLDKGFTPINFVFPHLPLPHYWKRDAA
QQKISETYMTEIARRRETGDIDENRDLIDSLLVNSTYKDGVKMTDQEIANLLIGVLMGGQHTSATTSAWFLLHLAEKPQLQDE
LYQEVLNALSGKGGNLDDLSYEDLQQMPLVNNTIKETLRLHMPLHSIFRKVVSPLVVPNTKYIVPRGHHVLVSPGYAHTNERF
YKDASDFNPHRWDESASTNDAGEVDYGFGKVSKGVSSSYLPFGGGRHRCIGEQFAYVQLGTILTTFVYNLKWKLANGKVPDVD
YTSMVTLPQHPAEIVWEKRDTCVIGGRHHHHHH

Protein sequence coverage is 88.32%

B. Mass spectrometry result of CpCYP51-6×His Y132F

Digestion of CpCYP51-6×His Y132F with trypsin. (The 'F' substitution at the 132 position is highlighted in red).

MALVDLALHGYNYFMTLSTLQQFGLLVFAPFIYNIIWQLLYSLRKDRVPLVFYWIPWVGSAVSYGQDPYGFFEQCREKYGDLF
SFVMLGRVMTVYLGPKGHEFVFNAKLSDVSAEDAYQHLTTPVFGKGVI**F**DCPNARLMEQKKFAKTALTTDSFRRYVPLIRGEI
LDYFTKSKVFNMKKQKSGVVDVLQSQPEITIFTASRSLLGEAMRKRFDASFAQLYADLDKGFTPINFVFPHLPLPHYWKRDAA
QQKISETYMTEIARRRETGDIDENRDLIDSLLVNSTYKDGVKMTDQEIANLLIGVLMGGQHTSATTSAWFLLHLAEKPQLQDE
LYQEVLNALSGKGGNLDDLSYEDLQQMPLVNNTIKETLRLHMPLHSIFRKVVSPLVVPNTKYIVPRGHHVLVSPGYAHTNERF
YKDASDFNPHRWDESASTNDAGEVDYGFGKVSKGVSSSYLPFGGGRHRCIGEQFAYVQLGTILTTFVYNLKWKLANGKVPDVD
YTSMVTLPQHPAEIVWEKRDTCVIGGRHHHHHH

Protein sequence coverage is 63.09%

Digestion of CpCYP51-6×His Y132F with chymotrypsin. (The 'F' substitution at the 132 position is highlighted in red).

MALVDLALHGYNYFMTLSTLQQFGLLVFAPFIYNIIWQLLYSLRKDRVPLVFYWIPWVGSAVSYGQDPYGFFEQCREKYGDLF
SFVMLGRVMTVYLGPKGHEFVFNAKLSDVSAEDAYQHLTTPVFGKGVI**F**DCPNARLMEQKKFAKTALTTDSFRRYVPLIRGEI
LDYFTKSKVFNMKKQKSGVVDVLQSQPEITIFTASRSLLGEAMRKRFDASFAQLYADLDKGFTPINFVFPHLPLPHYWKRDAA
QQKISETYMTEIARRRETGDIDENRDLIDSLLVNSTYKDGVKMTDQEIANLLIGVLMGGQHTSATTSAWFLLHLAEKPQLQDE
LYQEVLNALSGKGGNLDDLSYEDLQQMPLVNNTIKETLRLHMPLHSIFRKVVSPLVVPNTKYIVPRGHHVLVSPGYAHTNERF
YKDASDFNPHRWDESASTNDAGEVDYGFGKVSKGVSSSYLPFGGGRHRCIGEQFAYVQLGTILTTFVYNLKWKLANGKVPDVD
YTSMVTLPQHPAEIVWEKRDTCVIGGRHHHHHH

Protein sequence coverage is 93.97%

C. Mass spectrometry result of CpCPR-6×His

Digestion of CpCPR-6×His with trypsin.

```
MALDRLDLTVVIVLAVAVAAYFIKSQYFSKPESSGFLNTDTAGGNSRDILATLTKNHKNTLLLFGSQTGTAEDYCNKMSRELS
ARFGLKTMVADFADYDWDNFGDIKEDVLVFFIMATYGEGEPTDNAIEFVDFLDNEADTLSTLRFTVFGLGNSTYEFFNAIGRK
INEKLESKGAERFAEYGEGDDGQGTMDEDFLAWKDGVFDSLRNNLNLEEKELKYEPSLKLEIRDDLTIDDSSVSLGEPDKSYV
NTKAGTDLTKGPFDHSHPYLAPITKIKELFFTKERSCVHVEFDLSNSNLKYTTGDHLAIWPSNANEYVELFLKTFDLTEQRDV
VFDLKALDSTYQIPFPTPITYEAVVRHHLEISGPVSRQFFLSIAAFAPDEETKTKLTTVANDKQKYAEEVTHKKYNIADGLLY
FSNGKPWTKVPFEFLIENVQHFTPRYYSISSSSLSEKTHIDITAVVEAETESDGRVVTGVVTNLLKDVEINKNSSSDKPIVSY
DLKGPRNKFQNYKLPVHVRRSTFKLPSSSKTPIILVGPGTGVAPLRGFVRERVQQLKNGVNVGPSLLFYGCRNEDEDYLYRDE
WPQYAKELGESFELITAFSRANPNKKVYVQHKILEQAKKINQLLQDGGIIYVCGDASHMARDVQASFAKVLSQERGIELEKAA
ELIRSLKVQNRYQEDVWGGRHHHHHH
```

Protein sequence coverage is 90.14%.

Digestion of CpCPR-6×His with chymotrypsin.

```
MALDRLDLTVVIVLAVAVAAYFIKSQYFSKPESSGFLNTDTAGGNSRDILATLTKNHKNTLLLFGSQTGTAEDYCNKMSRELS
ARFGLKTMVADFADYDWDNFGDIKEDVLVFFIMATYGEGEPTDNAIEFVDFLDNEADTLSTLRFTVFGLGNSTYEFFNAIGRK
INEKLESKGAERFAEYGEGDDGQGTMDEDFLAWKDGVFDSLRNNLNLEEKELKYEPSLKLEIRDDLTIDDSSVSLGEPDKSYV
NTKAGTDLTKGPFDHSHPYLAPITKIKELFFTKERSCVHVEFDLSNSNLKYTTGDHLAIWPSNANEYVELFLKTFDLTEQRDV
VFDLKALDSTYQIPFPTPITYEAVVRHHLEISGPVSRQFFLSIAAFAPDEETKTKLTTVANDKQKYAEEVTHKKYNIADGLLY
FSNGKPWTKVPFEFLIENVQHFTPRYYSISSSSLSEKTHIDITAVVEAETESDGRVVTGVVTNLLKDVEINKNSSSDKPIVSY
DLKGPRNKFQNYKLPVHVRRSTFKLPSSSKTPIILVGPGTGVAPLRGFVRERVQQLKNGVNVGPSLLFYGCRNEDEDYLYRDE
WPQYAKELGESFELITAFSRANPNKKVYVQHKILEQAKKINQLLQDGGIIYVCGDASHMARDVQASFAKVLSQERGIELEKAA
ELIRSLKVQNRYQEDVWGGRHHHHHH
```

Protein sequence coverage is 94.49%

Figure S1 Identification of the recombinant proteins by mass spectrometry. Sequences identified on excision from SDS-PAGE gel bands are highlighted in grey.

References

1. Lamping E, Monk BC, Niimi K, Holmes AR, Tsao S, Tanabe K, et al. Characterization of three classes of membrane proteins involved in fungal azole resistance by functional hyperexpression in *Saccharomyces cerevisiae*. *Eukaryot Cell*. 2007;6:1150-65.
2. Monk BC, Tomasiak TM, Keniya MV, Huschmann FU, Tyndall JD, O'Connell JD, 3rd, et al. Architecture of a single membrane spanning cytochrome P450 suggests constraints that orient the catalytic domain relative to a bilayer. *Proc Natl Acad Sci U S A*. 2014;111:3865-70.
3. Sagatova AA, Keniya MV, Wilson RK, Sabherwal M, Tyndall JD, Monk BC. Triazole resistance mediated by mutations of a conserved active site tyrosine in fungal lanosterol 14alpha-demethylase. *Sci Rep*. 2016;6:26213.
4. Monk BC, Keniya MV, Sabherwal M, Wilson RK, Graham DO, Hassan HF, et al. Azole Resistance Reduces Susceptibility to the Tetrazole Antifungal VT-1161. *Antimicrob Agents Chemother*. 2019;63:e02114-18.
